# Stimulation Strategies for Improving the Resolution of Retinal Prostheses

**DOI:** 10.3389/fnins.2020.00262

**Published:** 2020-03-26

**Authors:** Wei Tong, Hamish Meffin, David J. Garrett, Michael R. Ibbotson

**Affiliations:** ^1^National Vision Research Institute, Australian College of Optometry, Carlton, VIC, Australia; ^2^Department of Optometry and Vision Sciences, Melbourne School of Health Sciences, The University of Melbourne, Melbourne, VIC, Australia; ^3^School of Physics, The University of Melbourne, Melbourne, VIC, Australia; ^4^Department of Biomedical Engineering, The University of Melbourne, Melbourne, VIC, Australia

**Keywords:** retina, retinal ganglion cell, electrical stimulation, stimulation resolution, retinal prostheses

## Abstract

Electrical stimulation using implantable devices with arrays of stimulating electrodes is an emerging therapy for neurological diseases. The performance of these devices depends greatly on their ability to activate populations of neurons with high spatiotemporal resolution. To study electrical stimulation of populations of neurons, retina serves as a useful model because the neural network is arranged in a planar array that is easy to access. Moreover, retinal prostheses are under development to restore vision by replacing the function of damaged light sensitive photoreceptors, which makes retinal research directly relevant for curing blindness. Here we provide a progress review on stimulation strategies developed in recent years to improve the resolution of electrical stimulation in retinal prostheses. We focus on studies performed with explanted retinas, in which electrophysiological techniques are the most advanced. We summarize achievements in improving the spatial and temporal resolution of electrical stimulation of the retina and methods to selectively stimulate neurons with different visual functions. Future directions for retinal prostheses development are also discussed, which could provide insights for other types of neuromodulatory devices in which high-resolution electrical stimulation is required.

## Introduction

**FIGURE 1:**
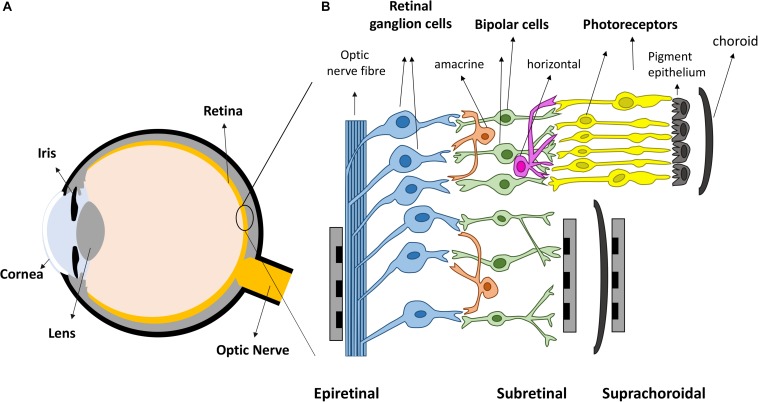
Retinal prostheses. **(A)** Schematic representation of the eye. Light enters the eye through cornea and is focused by the lens onto the retina. **(B)** The retina is mainly composed of three layers of neurons, photoreceptors, bipolar cells and retinal ganglion cells (RGCs), with horizontal and amacrine cells in between. Three different placements of retinal prostheses are under development. Epi-retinal implants are in contact with the RGC layer; sub-retinal devices are between the pigment epithelium and the remaining retina, and suprachoroidal devices are implanted between the choroid and sclera.

Vision is amongst the most vital tools for functioning in daily activities. In healthy eyes, light enters through the cornea and is focused by the cornea and lens, onto the retina, the light sensitive tissue lining the back of the eye (Figure 1A). The retina (Figure 1B) contains light sensitive photoreceptors, including rods and cones, which can then transduce the light into chemical and electrical signals. The signals are sent to other neurons in the retina, including bipolar cells and retinal ganglion cells (RGCs). RGCs have axons that collectively form the optic nerve and deliver neural signals to the central brain. The brain processes the signals in a series of complex ways to ultimately generate the sensation of vision.

Retinal degenerative diseases, including age-related macular degeneration (AMD) and retinitis pigmentosa (RP), are leading causes of major vision loss and blindness worldwide (Bourne et al., 2013). Approximately one in every 3,000–7,000 people is affected by RP (Ferrari et al., 2011) and over 8% of the population over 45 have evidence of macular degeneration (Wong W. L. et al., 2014). Both diseases lead to the loss of photoreceptor cells, thus depleting the ability of retinas to transduce light into useful visual signals. For both AMD and RP, currently available therapies normally only aim to slow down the death of photoreceptors, by providing nutritional supplements (Krishnadev et al., 2010) or through anti-vascular endothelial growth factor (anti-VEGF) injections (Ba et al., 2015) and lasers (Virgili et al., 2015), with limited available treatments for stopping the progression of the diseases or restoring vision. More recent treatments showing encouraging results include gene therapy and cell transplantation (Scholl et al., 2016). For both these therapies, several issues remain unresolved. Gene therapy currently suffers from limited recognized mutations for treatment (Hartong et al., 2006) and cell transplantation has difficulties with cell function and connectivity.

Over the last two decades, retinal prostheses that electrically stimulate surviving retinal neurons have emerged as a promising treatment for returning sight to the blind (Goetz and Palanker, 2016; Weiland et al., 2016). These devices can be categorized into three types depending on the location of the electrode arrays (Figure 1B). Epi-retinal devices have electrode arrays on top of the retina, in contact with the RGC layer. Sub-retinal implants are placed under the retina, closest to diseased photoreceptor layer. Suprachoroidal implants are between the sclera and choroid. Several devices have been implanted into human patients, such as Second Sight’s epi-retinal Argus II (Stronks and Dagnelie, 2014), Retina Implant AG’s sub-retinal Alpha AMS (Stingl et al., 2017), Bionic Vision Australia’s suprachoroidal devices (Ayton et al., 2014), and Pixium Vision’s epi-retinal IRIS II, and the most recent sub-retinal PRIMA. Most of the clinical results released by these consortiums have been positive: patients have reported the ability to detect light, categorize large objects from a list and even identify large letters (Zrenner et al., 2011; Humayun et al., 2012; Stingl et al., 2013; Ayton et al., 2014). Nevertheless, the visual resolution obtained from existing devices is very limited, meaning that even recognizing simple objects is challenging. Crucial abilities, such as facial recognition, are not yet possible. Snellen acuity is commonly used for describing visual acuity. A Snellen acuity of 20/20 represents normal vision, and 20/200 is defined legally blind. The best acuities reported in literatures so far from clinical trials are 20/1260 from Argus II (Humayun et al., 2012), 20/546 from Alpha-AMS (Stingl et al., 2017) and between 20/4451 and 20/21059 from BVA suprachoroidal devices (Ayton et al., 2014), all within legal blindness. The clinical results from retinal prostheses have been reviewed recently by Ayton et al. (2019).

Animal testing can evaluate and predict the performance of devices prior to clinical trials. Compared with *in vivo* testing, *ex vivo* experiments using explanted retinas are normally easier to perform, with more advanced electrophysiological approaches and have provided a large amount of important information to understand the performance of retinal prostheses. The knowledge gained from *ex vivo* experiments ranges from a better understanding of electrical stimulation, potential explanations of clinical observations, to the development of novel stimulation strategies. In this review, we first describe the current challenges in electrical stimulation of retinal neurons, which limit the performance of retinal prostheses. We then introduce the animal models commonly used, and recent advances in electrophysiological tools for retinal experiments. After this, progress in the last 5 years in improving the resolution of electrical stimulation of retinal neurons is summarized. Finally, we discuss the trends for the next generation of retinal prostheses, which could provide insights to future development and guide the design of other neuromodulation devices.

## Current Challenges and Limitations

The key challenges that inhibit visual function of retinal prostheses can be summarized as follows: (1) limited spatial resolution; (2) limited temporal precision; and (3) unselective activation of different visual pathways.

### Limited Spatial Resolution

Single electrode stimulation generates the perception of spots of light, referred to as phosphenes. However, patients often report phosphenes that are larger than the electrodes and distorted in shape. Ideally, stimulation of individual retinal neurons is desired to restore natural vision. There are over 1.5 million RGCs in the human retina (Harman et al., 2000) with the largest soma having a diameter of about 30 μm (Liu et al., 2017). Argus II devices stimulate with 60 electrodes, each of 200 μm in diameter (Dorn et al., 2013), Alpha AMS with 1600 electrodes, each of 30 μm (Stingl et al., 2017) and the BVA suprachoroidal devices with only 44 electrodes, each of 500 μm diameter (Ayton et al., 2014; Abbott et al., 2018). All of them are similar or far larger than the size of individual somas. There are several technical limitations to using higher density electrode arrays. For example, the impedance of electrodes increases when their size is reduced. High impedance electrodes require higher voltage stimulation drivers which consume more power. Many materials do not have suitable electrochemical properties to elicit neural activity within the safe charge injection limit.

Another common cause of low spatial confinement of activation is a gap between the electrode array and the surface of the retina. The electric field above a stimulating electrode rapidly spreads in a lateral direction with distance above the electrode resulting in a loss of spatial confinement. Epi-retinal devices are intended to stimulate RGCs, however large electrode-retina gaps after surgery have been reported (Gregori et al., 2018). Sub-retinal devices stimulate nearby inner retinal neurons and thereby take advantage of the natural signal processing by sending signals in the direction that a healthy retina would normally employ. For these devices, there is also potential separation between the inner retinal cells and the surface of the electrode array as degenerative retina often have a layer of debris as photoreceptors are replaced during degeneration. With suprachoroidal devices, the electrode/neuron separation is even larger – usually around 1 mm is expected.

Even when the placement of the electrodes is close and the size of the electrodes is comparable to the targeting neurons, there are other biological issues to be resolved. One critical problem is the activation of RGCs axon bundles (Fried et al., 2009), which is associated with patient reports of elongated phosphenes (Beyeler et al., 2019). This phenomenon occurs when electrodes not only stimulate the nearby neurons, but also errantly stimulate neurons from remote locations connected to the activated axons passing near the electrode.

### Limited Temporal Precision

In addition to localized activation, electrical stimulation with high temporal precision is required to replicate visual responses in retina. RGCs can be stimulated either directly by the electrode or indirectly through the retinal network. Network mediated stimulation may take advantages of the natural signal processing in the retina. In a subset of RGCs, their responses through network mediated stimulation were found to be similar to a natural light response, although delays of tens of ms were observed (Im and Fried, 2014). However, retinal remodeling can happen during degeneration (Jones and Marc, 2005), making it unclear if the natural signal processing function in retina is preserved or not. Compared with network mediated responses, the responses of RGCs to direct stimulation normally happen within a short delay (below 5 ms). However, the encoding of images based on the direct RGC responses requires sophisticated image processing techniques in order to account for the natural visual processing in retinal circuits.

Another problem limiting temporal performance is the loss of responses to high frequency repetitive stimulation which has been found in all types of retinal cells. In a healthy retina, photoreceptors can resolve repetitive frequencies of 20–50 Hz (Zrenner, 2013), leading to the RGCs firing at frequencies over 200 Hz (Koch et al., 2004). However, in most cases, retinal prostheses allow an image refreshment frequency of 5–20 Hz, and images “fade” after repetitive stimulation (Zrenner, 2013). Therefore, the loss of responses to high frequency repetitive stimulation may be one of the reasons for image fading.

### Unselective Activation of Different Visual Pathways

The third limitation for existing devices originates from the non-selective stimulation of the many visual pathways within retina. In general, RGCs can be classified as ON or OFF cells. The spike rates of ON cells increase when light illuminates the center of the cell’s receptive field, while the spike rates of OFF cells increase at light offset. In natural vision, ON and OFF cells in any patch of visual space are not activated simultaneously as light and dark patches are segregated. To date, more than 30 types of mammalian RGCs have been identified (Baden et al., 2016), each responsible for different aspects of visual information processing such as brightness, contrast, movement and color. Current retinal prostheses stimulate all types of retinal neurons in a similar manner without any preference, which is very different from the way that a healthy retina processes images. Approaches for selective activation of different RGC types are expected to significantly improve the vision restored.

## Experimental Methods

### Animal Models

The animal models that have been used for visual processing research range from salamander to primates (including humans). The most popular models for studying the responses of retinal cells to electrical stimulation are mice, rats, rabbits and monkeys. Mammalian species share similar types of neurons in retina, e.g., photoreceptors, bipolar cells and RGCs, along with horizontal and amacrine cells, which provide lateral interactions (Figure 1B). However, there are also some differences between species. For example, in humans and some other mammals such as monkeys and cats, the location of the highest acuity in the retina is a small region at the center of the visual field that has the highest density of RGCs (area centralis). In rabbits, the area of highest acuity in their retina is not a single, restricted region but an elongated zone running across the retina, referred to as the visual streak. In contrast, rodents have RGCs distributed more uniformly without an obvious area centralis or visual streak.

The terminology commonly used for referring to different types of RGCs in each species differs (Table 1). For example, RGCs with large somas, large dendritic sizes and large receptive fields are referred to as Alpha or A cells in rodents and cats, but can also be known as Y cells in cats. These cells are similar to so called brisk transient cells in rabbits and parasol cells in primates. These cells can be further classified into ON or OFF cells, although there are even more subgroups for A cells in rodents, including sustained ON, sustained OFF and transient OFF according to their light responses. RGCs with very small somas, small dendritic sizes and also small receptive fields are known as Beta or B cells in cats and rodents, but can also be known as X cells in cats. These cells are similar to so-called brisk sustained cells in rabbits and midget cells in monkeys. In primate, the midget cells are known to be the main vehicle for generating high-resolution vision, but the function of beta cells in rodents is less clear (Sanes and Masland, 2015). Similar with alpha (A) cells, beta (B) cells also have ON and OFF responses to light illumination.

**TABLE 1:** Commonly identified RGCs and their names in different animal models.

Animals	Mouse	Rat	Rabbit	Cat	Primate
Large soma and large dendritic field	Alpha cells	A cells	Brisk transient cells	Alpha cells/Y cells	Parasol cells
Small soma and small dendritic field	Beta cells	B cells	Brisk sustained cells	Beta cells/X cells	Midget cells

Despite the differences between retinas in rodents and primates, rodents are now the most popular species for research, in part due to their low costs and shorter breeding periods. Rodent animal models of retinal degeneration are also available, which are more relevant for studying retinal responses in terms of retinal prostheses. There are at least 15 mouse models of retinal degeneration with varying rates of photoreceptor loss, from a few days (rd1), to several months (rd10) (Chang et al., 2002). The commonly used rat models of retinal degeneration include Royal College of Surgeon (RCS), P23h and 344-ter rats (Goetz and Palanker, 2016). Photoreceptor degeneration is faster in RCS rats with complete death of photoreceptors and loss of light responses by the age of 90 days (P90) (Ryals et al., 2017). In the other two types of rats, the degeneration is slower, with light responses being found even at P500 in P23h rats (Sekirnjak et al., 2009). Depending on the stage of retinal degeneration of interest, different animal models have been used for different reasons. Abnormal spontaneous behaviors have been reported in degenerated retinas, e.g., RGCs tend to show low levels of background oscillation and bursts of spikes (Margolis and Detwiler, 2011). During electrical stimulation, such abnormal spontaneous activities lead to low signal-to-noise ratios (Choi et al., 2014). Several studies have also reported elevated thresholds for RGC stimulation in degenerated retinas (Jensen and Rizzo, 2009; Chan et al., 2011; Cho et al., 2016), although some others showed no significant differences (Sekirnjak et al., 2009; Cho et al., 2016). The differences observed between degenerated and healthy retinas further indicate the importance of using animal models with retinal degeneration for developing stimulation strategies for retinal prostheses.

### Electrophysiological Tools

**FIGURE 2:**
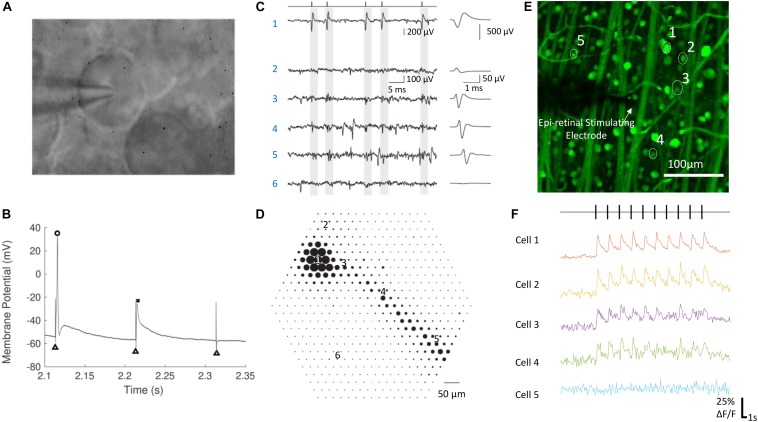
Electrophysiological techniques for recording neural responses in retina. **(A)** A RGC during whole cell patch clamping. The glass pipette electrode is in contact with the RGC’s soma. **(B)** The membrane potential of a RGC in response to electrical stimulation, with an action potential (circle), a spikelet (cross) and no response. Black triangles indicate the stimulation artifacts, which were at the time of stimulation. **(C,D)** Electrical image of a single RGC recorded by a multielectrode array. **(C)** Raw voltage traces (left) and the average waveforms (right) as a function of time recorded on the six electrodes indicated in **(D)**. The maximum absolute amplitude of average voltage deflections from **(C)** are shown for each of the 519 electrodes in the hexagonal array in **(D)**, indicated by the diameter of the dot plotted at each electrode location. Times of easily identified spikes recorded on Electrode 1 are identified as ticks in (**C**, left top). **(E,F)** Calcium imaging of a population of RGCs responding to electrical stimulation. The change of fluorescence intensities of five cells indicated in **(E)** to electrical stimulation are shown in **(F)**. **(A,B)** Are adapted with permission from Soto-Breceda et al. (2018). **(C,D)** are from Li et al. (2015). **(E,F)** Are adapted with permission from Tong et al. (2019b).

Several electrophysiological tools have been applied for recording the responses of retinal neurons to electrical stimulation (Figure 2). To retain the integrity of the retinal circuits, experiments are normally performed using whole-mount retina, kept in a perfusion chamber with oxygenated Ames’ medium at physiological temperatures between 33 and 37°C.

#### Patch Clamping

Patch clamping (Figures 2A,B) is one of the most commonly used techniques for intracellular recording of neural responses to electrical stimulation in retina. Whole-cell patch clamping allows simultaneous recording from multiple ion channels by measuring membrane potentials or ionic currents. The impact of synaptic activity and individual ion channels can be studied when using various synaptic or ion channel blockers. In some studies, loose patch clamping has been used, which is less invasive and does not damage the integrity of the cell membrane (Im and Fried, 2014, 2015, 2016; Lee et al., 2017; Im et al., 2018).

To record RGCs from whole-mount retinas, it is sometimes necessary to first reveal RGCs by making small holes in the inner limiting membrane (ILM), but this does little damage to the cells (Cloherty et al., 2012). To record the responses of inner retinal neurons, retinal slice preparations have been used to gain access to the cells (Margalit and Thoreson, 2006; Cameron et al., 2013). Disadvantages of retinal slices are that they sever lateral synaptic connections and suffer from significant current shunting around the tissue during stimulation (Margalit et al., 2011). In whole-mount retina, the patch clamping of bipolar cells was made possible by first peeling off the photoreceptors using filter paper (Walston et al., 2018). In another work, the patch clamping on inner retinal neurons was also achieved using sharp glass pipettes without removing any layer of the retina (Tsai et al., 2017a).

Although patch clamping can provide the most information about a single neuron, due to the difficulty of the technique, it only allows simultaneous recording of small numbers of neurons, and is delicate and time consuming, which requires a great deal of training, practice and experience.

#### Extracellular Recording

Extracellular recording is currently the only clinically viable method to measure retinal neuron signals. *Ex vivo*, it has been performed using either single sharp electrodes made of metals or carbon fibers, or multielectrode arrays (Figures 2C,D). Compared with intracellular recording, the signal-to-noise ratio from extracellular recording is lower, so it is more difficult to remove the artifacts arising from electrical stimulation. Recordings from single electrodes can only record from a small number of single neurons, while population information can be obtained using multi-electrode arrays. With the latest multielectrode array systems, it is possible to simultaneously record and classify recordings from more than 1,700 RGCs in a single experiment, using the high spatial and temporal spiking activities collected from the recording system (Tsai et al., 2017b). Such recording, at subcellular resolution, is termed electrical imaging, and its principle and application has been reviewed by Zeck et al. (2017).

#### Optical Imaging

Optical imaging using activity sensitive fluorescent dyes, mainly calcium imaging (Figures 2E,F), is another useful electrophysiological tool for studying the activities of neurons in retina. The dyes are first introduced into target neurons and the change of fluorescence intensity is used to infer neural activities, such as action potentials. Calcium imaging techniques for studying neural activity has been reviewed previously (Grienberger and Konnerth, 2012). The advantages of optical imaging include easy identification of soma locations and an absence of an electrical artifact, both of which reduce the burden of data analysis compared with electrical recording. Although recording of single action potentials with calcium imaging has been demonstrated (Smetters et al., 1999), this has rarely been demonstrated in the retina. Optical imaging is slow and therefore yields low temporal resolution recordings compared with electrical recording. One limitation lies in the low imaging frame rates available on most microscopes (normally around 10–20 Hz), significantly lower than sampling frequencies used during electrical recording (∼10–40 kHz). In addition, the fluorescence intensity of the activity indicators needs some time to decay to their background levels following neural activity and, depending on the indicator type and strength of the neural activity, the decay may take up to several seconds.

Several techniques have been reported for large-area loading of retinal cells with calcium indicators. Behrend et al. (2009) first reported the loading of RGCs in whole-mount retinas by immersing the optic nerve stumps in dye solution, but the method failed in adult mammal retinas. Multicell bolus loading (Borghuis et al., 2011) using membrane permeable indicators was reported but uniform staining was difficult. Other methods that successfully stained RGCs in mammalian retinas include electroporation (Baden et al., 2016), dye incubation after dissolving the ILMs (Cameron et al., 2016), direct dye injection into the optic nerve (Tong et al., 2019a), and transduction with genetically encoded calcium indicators through adeno-associated viral vectors (Weitz et al., 2015). To reveal the activities of degenerated photoreceptors to electrical stimulation, incubation of retina with cell permeable dyes has been reported (Haq et al., 2018).

## Recent Progress

### Electrical Stimulation of Retinal Neurons

Electrical stimulation of retinal neurons can be delivered intracellularly or extracellularly. Intracellular stimulation works by directly injecting current into the cells, normally through patch clamping electrodes. No clinical application is currently available for intracellular stimulation. Nevertheless, intracellular stimulation is a useful approach for characterizing the intrinsic properties of neurons (Wong et al., 2012; Hadjinicolaou et al., 2016). Without contribution from the network, intracellular stimulation simplifies the study by focusing on the properties of the recorded neurons and avoiding the complexity of extracellular stimulation, in which the placement of the electrode plays a significant role.

**FIGURE 3:**
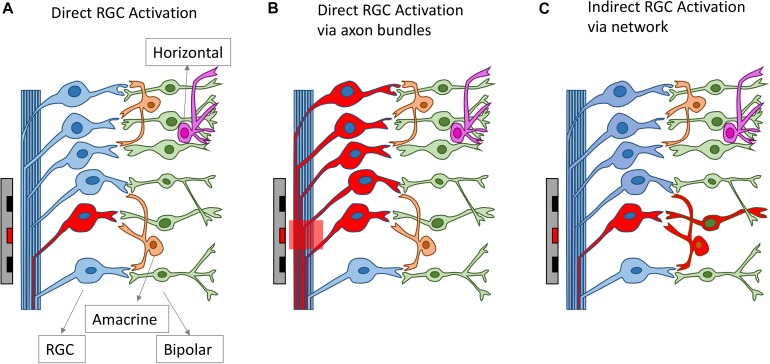
Different routes for RGC activation arising from electrical stimulation. The electrodes and the neurons activated are drawn as red. RGCs can be activated directly from electrical stimulation **(A)**, via axon bundles **(B)** or indirectly via the retinal network **(C)**.

All clinical neural implants operating today use extracellular stimulation, which works by depolarizing cells in an electric field, instead of directly injecting current into the cells (Rattay, 1999; Meffin et al., 2012). In the most common mode of RGC stimulation, a non-uniform electric field is required. The non-uniform electric field causes charges to redistribute across the membrane of an axon or dendrite and concentrates them at the point where the gradient of the electric field is the greatest along the fiber. Stimulation of bipolar cells is most common through depolarization caused by charge accumulation at synaptic terminals, which can occur even in a uniform electric field directed across the cell (Werginz and Rattay, 2016). Firing of action potentials can be initiated when the membrane depolarization exceeds a threshold. The charge redistribution may happen on the membranes of axons, somas and dendrites, which all contribute to the depolarization of the retinal neurons. For RGCs, experimental evidence indicates that the axon initial segment (AIS), which is located at the proximal end to the soma and contains a high density of sodium channels, is the most sensitive area for activation (Fried et al., 2009). The AIS has the lowest activation threshold, followed by other axonal sections and the soma, with the dendrites exhibiting the highest threshold to electrical stimulation (Fried et al., 2009; Tsai et al., 2012). In addition to RGCs, extracellular stimulation can also lead to the activation of other retinal neurons, including bipolar cells and photoreceptors in healthy retinas, which will then activate RGCs through neuro-transmitters, in the same way that the retina processes visual stimuli. With extracellular stimulation, RGCs can be activated mainly through one of three routes (Figure 3): (1) direct activation through the AIS; (2) direct activation via axon bundles; (3) indirect activation via the retinal network. How RGCs are activated can determine the spatial and temporal resolution of electrical stimulation, which will be discussed in sections Spatial Resolution and Temporal Resolution.

**FIGURE 4:**
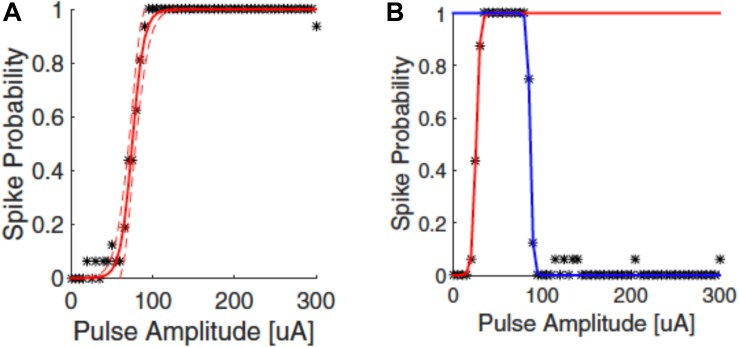
Upper threshold phenomenon in RGC stimulation. Depending on the stimulation parameters, RGCs may show upper threshold phenomena. Normally, RGC response efficacy increases with stimulus strength (pulse amplitude) and then saturates, as shown in **(A)**. When exhibiting the upper threshold phenomenon **(B)**, the response efficacy drops when stimulus strength exceeds a certain amount. Adapted with permission from Meng et al. (2018).

The responses of neurons to electrical stimulation, for a fixed pulse duration, normally follows a sigmoidal function: the response efficacy increases with stimulus strength (current, voltage or charge), then reaches a maximum and saturates (Tsai et al., 2009; Meng et al., 2018; Figure 4A). The stimulus strength associated with 50% response efficacy is usually defined as the threshold of activation. Lowering the stimulation threshold is very important for retinal prostheses as larger thresholds consume more power and may exceed the safe limit of the electrode materials or tissue. As neurons are activated due to the electric fields generated by the electrodes, the stimulus effectiveness is greatly influenced by electrode size and the distance between electrode and neuron. Research has also indicated that stimulus effectiveness can be greatly influenced by various stimulation parameters. For example, Weitz et al. (2014) found that, for biphasic pulses, the currents required for RGC activation decreased as the interphase durations increased. Hadjinicolaou et al. (2015) and Jalligampala et al. (2017) proposed strategies for searching the most efficient stimulation parameters for RGCs activation.

Recent years have also revealed an interesting observation known as the upper threshold phenomenon (Figure 4B) in RGC activation, i.e., a drop of response efficacy instead of saturation when stimulus strength exceeds a certain amount. The upper thresholds phenomenon was first observed by Boinagrov et al. (2012), and then reported again in several other studies (Barriga-Rivera et al., 2017; Guo et al., 2018; Kotsakidis et al., 2018; Meng et al., 2018). Meng et al. (2018) found that 20/21 cells exhibited the upper threshold phenomenon when sufficiently high charge was injected. However, from modeling they observed different results between monophasic and biphasic stimulation. While the upper threshold in the soma was observed in simulation for both types of stimulation, the action potential in the distal axon was blocked with monophasic stimulation but not with biphasic pulses. However, the upper threshold phenomenon with biphasic stimulation has been reported *in vivo* by Barriga-Rivera et al. (2017) that the recorded spike rates decreased in some channels with high amplitude stimulation. This indicates that, different from Meng et al. (2018), the upper threshold phenomenon may also happen in the RGC axons by biphasic stimulation. The potential mechanisms for the upper threshold phenomenon have been discussed in detail by Guo et al. (2019). According to their discussion, the sodium channel kinetics in RGCs may play the major role in the upper threshold phenomenon.

### Spatial Resolution

To confine electric fields generated by stimulating electrodes, attempts have been made to shape the electric fields with current focusing, e.g., replacing the remote return electrodes with local returns (Abramian et al., 2011, 2014; Flores et al., 2016, 2018; Matteucci et al., 2016; Fan et al., 2019; Tong et al., 2019a). Different local return configurations have been reported and compared, including connecting several stimulating electrodes as the return (Abramian et al., 2011, 2014; Matteucci et al., 2016), and specially designing a ring-shaped electrode surrounding the stimulating electrode as the local return (Flores et al., 2016, 2018; Fan et al., 2019; Tong et al., 2019a). A more detailed discussion about different local return configurations can be found in section Simultaneous Stimulation. Overall, the local returns have been shown to confine the activation of RGCs to a certain extent. For example, Fan et al. (2019) reported that the return provided by six neighboring electrodes can enhance the capability of 10 μm epi-retinal electrodes to activate cells near (<30 μm) the central electrode. However, this study focused only on the parasol cells, which are large in size. It remains unknown how the impact of a local return would affect spatial resolution when considering other neurons in the study, in particular the midget cells which are believed to be responsible for high acuity vision in primates. Furthermore, axon bundle activation was also neglected in this study, which is another main origin of RGC spread for epi-retinal stimulation. In another investigation, Tong et al. (2019a) compared the effect of return configurations for sub-retinal stimulation and showed different results depending on pulse durations and retinal degeneration. In the healthy retina, local returns were more effective in confining RGC activation when 0.1 and 0.2 ms pulses were used in comparison with 0.5 ms pulses. However, in the degenerated retina the RGC activation patterns were similar between two return configurations, regardless of the pulse durations.

Both simulation and experimental results also indicate that more charge or current will be required for neural stimulation when using local returns, due to the decrease of electric field intensity (Tong et al., 2019a). The elevated thresholds will lead to larger power consumption and a greater charge requirement for the electrodes. To reduce thresholds whilst minimizing a loss of electric field confinement, Flores et al. (2018) proposed local returns in conjunction with pillar structured electrodes. The pillar electrodes reduced the distance between the stimulating electrodes and the target neurons. Experimentally (Ho et al., 2019), they demonstrated *in vivo* that 10 μm tall pillars with 55 μm pixels can lead to grating acuities of 48 ± 11 μm, which matches the linear pixel pitch of the hexagonal arrays they used. When converting the value into human visual acuity, the result is close to 20/192, which is just within the legal blindness threshold of 20/200. Following these studies, they also proposed honeycomb-shaped electrodes for sub-retinal stimulation (Flores et al., 2019), where the stimulating electrodes sit within a deep honeycomb well, the walls of the well acting as the local returns. Experimentally (Flores et al., 2019) they demonstrated that the inner retinal cells migrated into the 25 μm deep wells after 5 weeks of implantation. No experimental stimulating results have been published using such arrays, but from simulation, the visual acuity is expected to be better than 20/100.

Merely reducing the size of the electric field is sometimes insufficient to confine the activation of retinal cells. As mentioned above, the epi-retinal stimulation using electrodes as small as 10 μm can also lead to a large spread of RGC activation due to axon bundle stimulation (Behrend et al., 2011). In another study, Grosberg et al. (2017) found that only 45% of electrodes, also 10 μm in diameter, can stimulate individual RGCs using current amplitudes below threshold for axon bundle activation. Therefore, the activation of axon bundles has been identified as one the main sources of the spread of retinal cell activation. The phenomenon is observed for both epi- and sub-retinal stimulation (Tong et al., 2019b).

**FIGURE 5:**
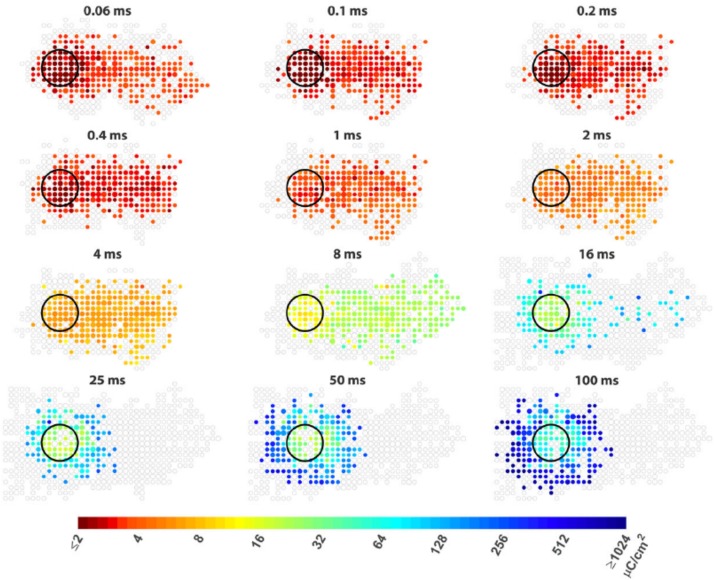
Improving the spatial resolution of electrical stimulation. The spatial threshold maps of RGC responses to epi-retinal stimulation with different pulse durations. The stimulating electrode is shown as black circles. Each colored dot represents the average threshold charge density needed to stimulate cells at its location. The area containing cells that did not response to stimulation are shown as unfilled gray dots. The dot sizes indicate the number of cells. Axon run from right (somas) to left (optic disc). The colored dots at right indicate somas of the cells whose passing axons are activated by the electrode. Figure adapted with permission from Weitz et al. (2015).

Strategies for avoiding axon bundle stimulation can be divided in to two routes. The first of these involves bypassing axon bundle stimulation by indirectly stimulating RGCs (Haq et al., 2018; Weitz et al., 2015). Weitz et al. (2015; Figure 5) demonstrated, via calcium imaging, that epi-retinal stimulation using both 24 ms biphasic square pulses and 20 Hz sine waves could effectively confine the RGC activation pattern because that type of stimulation primarily stimulates cells in the inner nuclear layer (inner retinal neurons) which, in turn, activate RGCs via the retinal network. Haq et al. (2018) studied sub-retinal stimulation using 1 ms voltage pulses, which were found effective for the activation of both degenerated cone photoreceptors (d-Phr) and inner retinal neurons. They showed that the 3 μm tip diameter electrodes used in the study mostly stimulated d-Phr about 60 μm, and RGCs about 160 μm from the electrodes. By applying gap junction blockers, they found that both the spread of d-Phr and RGC activation could be confined. However, there are other studies reporting different results about the spatial resolution of RGC activation resulting from network stimulation. Hosseinzadeh et al. (2017) studied the spatial extent of epi-retinal stimulation by focusing on the network responses from the electrodes. They found that the network responses can also spread to a large area 300–1034 μm away from the electrodes even when the electrodes were as small as 10 μm. For sub-retinal stimulation, Tong et al. (2019a, b) also reported the spread of RGC activation when using 25 ms long pulses that mainly stimulated inner retinal neurons. One possible hypothesis (Tong et al., 2019a) is that network stimulation could lead to the activation of RGC dendritic fields, which could be as large as 500 μm in certain RGC types. The discrepancy could be due to the different techniques used for recording. As discussed in section Electrophysiological Tools, typical multilelectrode arrays provide limited spatial coverage and/or resolution and the activated neurons could be out of the recording region or lie between the recording electrodes. On the other hand, calcium imaging may not have sensitivity high enough to detect single spikes and may not record every activated neurons.

Potential problems with network stimulation include its low temporal resolution (section Temporal Resolution), and the relatively higher charge thresholds required for neuron activation. With long pulses, the charge injection capacities for activation can be larger than 1 mC/cm^2^ (Weitz et al., 2015; Tong et al., 2019a), which will require the use of electrode materials with much large charge injection capacity than conventional materials such as platinum (charge injection capacity ∼150 μC/cm^2^). The larger charge required also consumes more power and leads to more heat generation.

The other strategy aimed at selectively activating RGCs within or near the electrodes is by increasing the difference between axon bundle and RGC soma activation (Chang et al., 2019; Tong et al., 2019a). For both epi-retinal and sub-retinal stimulation, ultrashort pulses (shorter than 0.15 ms in Chang et al., 2019, and shorter than 0.1 ms in Tong et al., 2019a) were demonstrated to be effective at avoiding axon bundle stimulation. Esler et al. (2018a) proposed to simultaneously stimulate multiple electrodes aligned with the axon bundles to minimize the bundle activation. The proposal was based on the fact that the excitable parts of RGC are the AIS. AIS are located in the RGC layer with random orientations, but the overlying axons are packed together as mostly parallel fibers. The simultaneous stimulation of electrodes parallel to the axons in the nerve fiber layer flattens the extracellular potential along the length of the axon, thus minimizing axon activation.

Human trials have shown that some patients see halo-shaped stimulation patterns from single electrodes (Humayun et al., 2003). There are also studies that provide insights to explain these halo-shaped phosphenes. Eickenscheidt and Zeck (2014) showed that the neurons with the lowest thresholds were at the edge of the stimulation electrode, where the gradient of the extracellular electric field is maximal. In another study (Barriga-Rivera et al., 2017), the halo-like phosphene shapes were explained using the upper threshold phenomenon. Here they found that neurons close to the stimulating electrodes were inhibited at amplitudes lower than the neurons far from the stimulating electrodes. The halo-shapes could also originate from network stimulation: Tong et al. (2019a) showed that long pulses tend to activate neurons further away from the stimulating electrodes compared to the RGCs within the electrodes.

### Temporal Resolution

High quality vision restoration requires the control of retinal neural activities with precise timing, on similar time scales to normal visual responses. There has been research demonstrating electrical stimulation of RGCs with temporal patterns resembling light-evoked spike trains (Jepson et al., 2014b; Wong R. C et al., 2014). For example, Jepson et al. (2014b) reported reproduction of the temporal spiking sequence to visual responses in populations of macaque monkey ON parasol cells. Similar results were reported by Wong R. C et al. (2014) in cat brisk transient cells. However, in both studies only limited types of cells were recorded and analyzed; it remains unclear how the electrical stimulation of other cell types, in particular the midget cells responsible for high acuity vision, could replicate visual responses.

A good understanding of the temporal patterns of all types of retinal neurons following electrical stimulation could inform the design of stimulation strategies for retinal prostheses. In general, responses originating in RGCs show short latencies (<5 ms), those originating in the inner nuclear layer show medium latencies (3–70 ms), and the responses originating in photoreceptors show long latencies (>40 ms) (Boinagrov et al., 2014). The activation of RGCs through direct- or network-mediated stimulation depends on the electrode location, pulse duration and pulse polarity. For example, Boinagrov et al. (2014) showed that monophasic cathodic epi-retinal stimulation with short pulses (below 0.5 ms) tends to directly stimulate RGCs, while long monophasic anodic pulses (above 10 ms) with electrodes in the outer plexiform layer showed optimal selectivity for network-mediated stimulation.

The response latencies of RGCs from direct stimulation exhibit a U-shape in relation to current amplitudes (Boinagrov et al., 2014; Meng et al., 2018). Compared with direct stimulation, the network mediated responses of RGCs are normally slower and exhibit a variety of temporal response patterns depending on the types of the cells and the stimulus parameters. Im and Fried (2015) compared light and network mediated electrical responses in different types of RGCs from wild type rabbits. They showed that the response patterns to a single pulse stimulus varied between ON and OFF brisk transient or brisk sustained cells, which can also be used to infer the type of neuron recorded. The network mediated electrical responses from ON cells could resemble their light responses much better than OFF cells. Also, the stimuli that activated photoreceptors yielded better correlations than those activating bipolar cells. In a following study (Im and Fried, 2016), they examined the network-mediated responses to repetitive stimulation and also found differences between ON and OFF cells. In both brisk transient and brisk sustained ON cells, they showed a reset phenomenon, in which each new stimulus elicited a brief burst of spikes. In contrast, OFF cells did not exhibit a reset in their responses; the responses to subsequent stimuli were diminished. Later, they demonstrated that varying stimulus durations (Im et al., 2018) could differentially modulate the responses between ON and OFF cells, providing a potential strategy for selective stimulation of different RGC types (see section Selective Activation). There are some further reports about the effects of varying stimulation patterns, such as duration, rate, current amplitudes, and waveform shapes (Im et al., 2018; Werginz et al., 2018; Lee and Im, 2019). This research mainly used wild-type animals, however, for the design of retinal prostheses, how cells in degenerated retinas respond to electrical stimulation is more relevant. Lee et al. (2017) found that the network mediated responses of ON alpha RGCs in rd10 mice showed trial-to-trial variability and the variability increased over the course of retinal degeneration. More research needs to be done in the future to understand the impact of retinal degeneration.

In addition to RGCs, there is research recording directly from other types of retinal neurons. A survey of electrically evoked responses over different current amplitudes and pulse durations were performed by Tsai et al. (2017a). In this study, they found differences among 21 cell types in response to electrical stimulation, a finding which may enable preferential recruitment of certain cell types. Walston et al. (2018) studied ON-type bipolar cells in both normal and degenerated mouse retina, and reported desensitizing responses to repeated stimulation and the upper threshold phenomenon.

As previously mentioned (section Current Challenges and Limitations), fading is one critical problem in retinal prostheses and has been found to be associated with the desensitized responses of retinal cells to repetitive stimulation. The desensitization phenomenon has been observed experimentally in both direct and network mediated responses of RGCs. For network mediated responses, in the low frequency range (below 10 Hz), Im and Fried (2016) showed desensitization in OFF RGCs, but not in ON RGCs. However, Walston et al. (2018) observed desensitizing responses in ON bipolar cells at frequencies greater than 6 Hz. The cut-off frequencies of direct responses of RGCs vary among morphological types (Hadjinicolaou et al., 2016), and differed between intracellular and extracellular stimulation (Kotsakidis et al., 2018). One possible mechanism of desensitization in direct responses of RGCs is a lack of sodium channel deinactivation (Tsai et al., 2011). Other studies have emphasized the existence of electrical currents in the retina, like axo-axonal gap junctions, which could cause an inhibition in the neuron, thus preventing it from generating full action potentials (Soto-Breceda et al., 2018).

**FIGURE 6:**
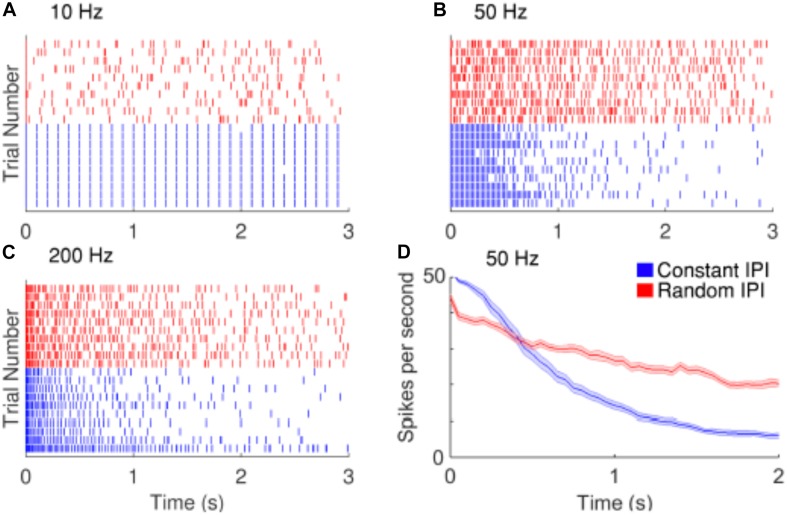
Improving the temporal resolution of electrical stimulation. RGCs show desensitizing responses to repetitive stimulation with high frequencies. **(A–C)** Raster plots of the responses of a cell to 10, 50, and 200 Hz electrical stimulation. Red and blue rasters represent action potentials in response to random and constant inter-pulse interval (IPI) stimulation, respectively. **(D)** The average spike rate decreases at a slower rate when stimulated with random IPI. Figure is from Soto-Breceda et al. (2018).

Strategies have been proposed to reduce the decay of RGCs responses during repetitive stimulation. Soto-Breceda et al. (2018) proposed the use of electrical pulses with irregular time intervals between them to replace periodic pulses that are normally used in retinal prostheses (Figure 6). They found that the random interpulse intervals could lead to lower adaptation rates than stimulation with constant intervals at frequencies above 50 Hz. In another study, Sekhar et al. (2016) analyzed the network mediated responses of RGCs to stimulation at 25 Hz, which would typically induce strong fading. As the retinal neurons could respond to sequences of subthreshold stimulation, they suggested the use of subthreshold sequences to minimize the fading problem.

### Selective Activation

There have been some encouraging results about selective activation of individual neurons. For example, Jepson et al. (2013) first demonstrated that it is possible to stimulate a single RGC without activating neighboring cells. Selectivity was improved by the use of local returns (Fan et al., 2019). However, there are several limitations in these studies for retinal prosthesis application. First, the electrodes used for stimulation were very small, with diameters around 10 μm, and in direct contact with the retina surface (epi-retinal stimulation). Clinically available devices use electrode sizes much larger; and there is usually some separation in space between the target neurons and the electrodes (Gregori et al., 2018). Secondly, these studies recorded and analyzed limited number of neurons within certain cell types. Jepson et al. (2013) examined the responses from midget, parasol and bistratified ganglion cells in the primate retina, while Fan et al. (2019) only examined the parasol cells. It is possible that other neuron types were also activated but not recorded or analyzed. Third limitation lies in the multielectrode array technique they used for recording, that activated neurons could be out of the recording region or lie between the recording electrodes.

**FIGURE 7:**
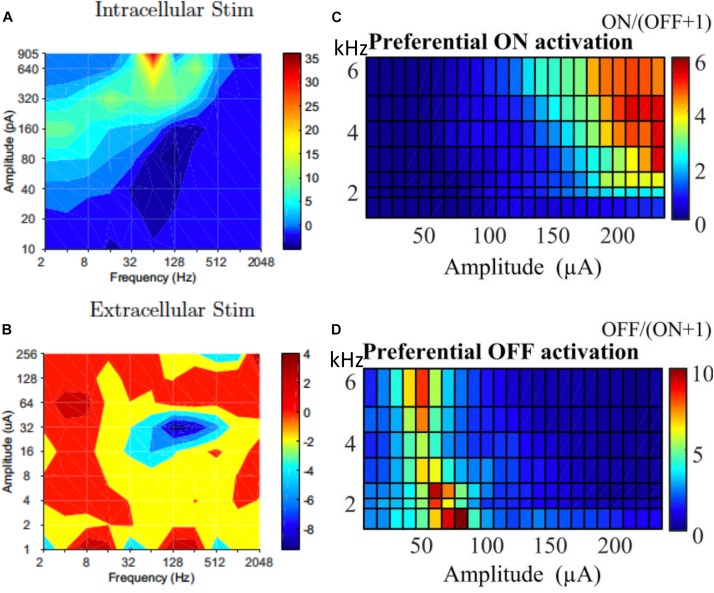
Selective activation of different types of RGCs. Contour map showing difference between ON and OFF RGC population response to intracellular **(A)** and extracellular **(B)** sinusoidal stimulation at frequencies of 2–2048 Hz. Colorbars show spiking rate in response to stimulation. High frequency (1–6 kHz) stimulation is used for selective ON and OFF RGC activation. **(C)** ON cells could be preferentially activated with high current amplitude and high frequencies. **(D)** OFF cells preferred lower stimulation amplitudes across all tested frequencies. **(A,B)** Is adapted from Kotsakidis et al. (2018). **(C,D)** Is adapted from Guo et al. (2018).

While selectively stimulating individual RGCs may be too challenging for current technologies, preferential activation of selective types of RGCs can also be beneficial to the quality of the vision restored. In response to intracellular stimulation, RGCs showed similarities within the same morphological types (Wong et al., 2012; Hadjinicolaou et al., 2016; Zehra et al., 2018). The difference between morphological types indicates the possibility to selectively stimulate RGCs with intracellular stimulation. However this may have limited relevance to extracellular stimulation. Kotsakidis et al. (2018) examined the optimal range of combinations of current amplitude and frequencies (2–2048 Hz) that preferentially activate ON over OFF RGC population responses (Figures 7A,B), and they found the optimal ranges were very different between intracellular and extracellular stimulation.

In addition to the work of Kotsakidis et al. (2018), there are several other studies that demonstrated the successful selective activation of ON or OFF RGCs. Similar to that of Kotsakidis et al. (2018), some of these studies focused on optimizing the current amplitudes and stimulation frequencies for each cell type. Cai et al. (2013) pioneered the work using high frequency (1 kHz) biphasic stimulation. They found that the OFF-brisk transient cells in rabbits could only be activated with a medium range of current amplitudes, but the ON-OFF directionally selective cells maintained strong spiking when much higher current amplitudes were applied. Twyford et al. (2014) used 2 kHz stimulation with amplitude modulation using a slower envelope and successfully modulated the activities of ON and OFF cells in a differential manner. Guo et al. (2018) then systematically studied ON and OFF responses to high frequency stimulation (>1 kHz) with constant amplitudes (Figures 7C,D). With synaptic blockers, ON cells were preferentially activated at relatively higher stimulation amplitudes (>150 μA) and frequencies (2–6.25 kHz), however, OFF RGCs were activated by lower stimulation amplitudes (40–90 μA) across all tested frequencies. The mechanisms underlying differential responses of ON and OFF cells have not been revealed experimentally but may be due to different ionic currents present in ON and OFF cells and different cell morphologies, as illustrated computationally (Guo et al., 2014, 2018; Kameneva et al., 2016).

Another strategy for selective stimulation is based on different pulse durations, as reported in Im et al. (2018) and Lee and Im (2019). Both works studied the network mediated responses of RGCs. In Im’s work (Haq et al., 2018), they found the activities of ON cells decreased significantly when the pulse duration increased. However, the changes of OFF cells to pulse duration were more modest. Lee and Im (2019) also found that ON cells are more sensitive to the change of current amplitude. Both works suggested that it is possible to bias the activation in favor of ON cells. However, it is unclear whether the differences between ON and OFF cells caused by network-mediated activation will remain in degenerated retina.

The third strategy investigated the impact of electrode configurations. Yang et al. (2018) showed that with synaptic blockers, ON RGCs showed higher thresholds than OFF RGCs for epi-retinal stimulation. Furthermore, the difference was enhanced when placing the stimulating electrodes away from the axon. However, the precise control of the stimulating electrode location is difficult during implantation, therefore its clinical application is challenging. With local returns, Fan et al. (2019) also showed selective activation of ON or OFF parasol cells. Guo et al. (2017) proposed the use of multiple stimulating electrodes, with a primary electrode near the target neurons and a bipolar return electrode pair near the optic disc. With their strategy, the propagation of OFF cells was blocked according to the computer simulation.

The last strategy determines the optimal waveforms for ON and OFF cell activation using spike-triggered analysis. Spike-triggered analysis was first used to determine the receptive fields of RGCs to visual stimuli, and has been used in recent years for studying the temporal and spatial electrical receptive fields of RGCs. A spatial electrical receptive field consist of the spatial arrangement of electrodes capable of stimulating a cell to spike, while a temporal electrical receptive field consist of the sequence of pulses the affected spike stimulation. The recent progress in spike triggered analysis for retinal stimulation is summarized in Rathbun et al. (2018). Sekhar et al. (2016) first reconstructed the temporal electrical receptive fields of RGCs in wild type mice and found that the waveforms were different for ON and OFF cells. After further analysis (Sekhar et al., 2017), they showed the waveforms had different polarities. ON cells tended to show waveforms with short-latency upward deflections, while OFF cells were correlated to short-latency downward deflections. Ho et al. (2018) obtain similar results, and showed that they could be attributed to photoreceptor response and it differential impact on ON and OFF bipolar cells. Although different receptive field polarities were also observed in the degenerated retina, it was not possible to identify the cell type. Comparing the waveforms between healthy and degenerated retinas, they found significant differences between the latencies and widths of the waveform deflections, which were shorter and narrower in degenerated retina. Similar results were also reported in Ho et al. (2018) for sub-retinal photovoltaic stimulation. One hypothesis about the presence of two response polarities in the degenerate retina relates to the depolarization of the rod bipolar cells (Ho et al., 2018). The depolarization of the rod bipolar cells would lead to the activation of ON RGCs but inhibition of OFF RGCs.

While most of the existing research aims at selective stimulation of ON vs. OFF cells, little research has been reported to preferentially activate cells in a broader range of cell types. One reported study showed preferential activation of brisk transient cells in rabbits (Im and Fried, 2015). They found anodic pulses could selectively activate brisk transient cells but not in brisk sustained cells. The same group later also found that the duration strength curves were different for brisk transient and brisk sustained cells (Im et al., 2018).

### Multielectrode Stimulation

Research concerning retinal cell responses to single electrode stimulation has provided the community with important information contributing to a deeper understanding of stimulation mechanisms and performance. However, to translate electrical stimulation to useful visual information in patients, it is necessary to stimulate multiple electrodes to create 2-D patterns, either simultaneously or in sequence. The knowledge collected from single electrodes can inform the stimulation strategies for multielectrode stimulation. The resolution of the percepts reproduced depends on the selection of electrodes and stimulation parameters.

#### Sequential Stimulation

Sequential stimulation was performed by Shah et al. (2019), in which each electrode was stimulated in series, at a rate expected to be faster than the integration time of visual perception. They first created a response library by recording the RGC responses to individual electrode stimulation. Then to reconstruct the image, they stimulated the electrodes one by one. In each time frame, the stimulation electrode was determined using a greedy algorithm. This algorithm was built on the collected library and aimed at minimizing the difference between the accumulated stimulation pattern and the target. They further found the efficacy of image reconstruction to be better if they limited the stimulation library to the most frequently chosen electrodes. In this work, the error between the activation patterns and the targets monotonically decreased with the number of stimulation patterns delivered, but saturated after 4,000. However, to stimulate 4,000 electrodes in series at a frequency of 10 kHz requires 400 ms, which is much longer than the likely integration times in the brain, which is expected to be tens of ms.

#### Simultaneous Stimulation

In clinical retinal implants, when neighboring electrodes are stimulated simultaneously, phosphenes tend to overlap, resulting in spatial resolution that is poor compared to the density of electrodes. This is a consequence of the spread of current from the stimulating electrode to areas underlying adjacent electrodes, resulting in an increase in the area of retinal activation encompassing several electrodes. At first, simultaneous stimulation on neighboring electrodes may seem likely to exacerbate this problem. However, some stimulation strategies propose to make use of simultaneous stimulation to focus, shift or otherwise shape retinal activity to overcome the problem and thereby improve spatial resolution toward the limits imposed by electrode density.

**FIGURE 8:**
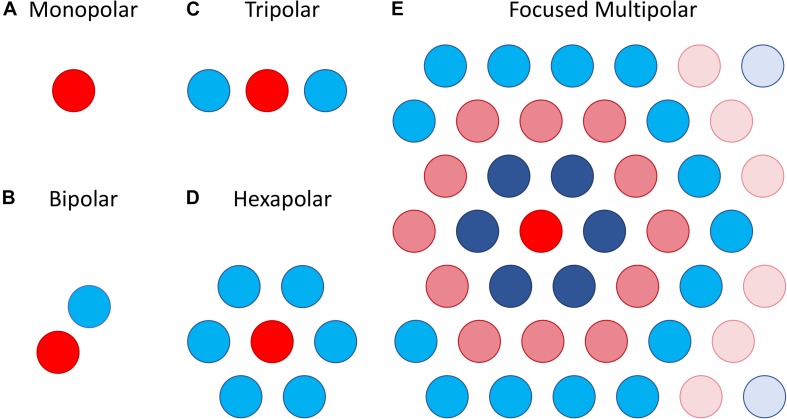
Illustration of different electrode configurations for current focusing. **(A)** Monopolar stimulation is represented as a single stimulating electrode. **(B–D)** Bipolar, tripolar and hexapolar stimulation with negative weights on the other one, two or six surrounding electrodes as these are used as return electrodes. **(E)** Focused multipolar weights often presented as concentric rings of alternating positive and negative currents radiating from a central electrode. Each ring aims to cancel out the spread of current produced by the former.

The most straightforward approach attempts to focus the area of retinal activation to just the area immediately under or around a stimulating electrode by using the adjacent electrodes as current sinks. This can be done using bipolar, tripolar or multipolar electrode configurations (Cicione et al., 2012; Figure 8). The rationale behind such approaches is that they contain the spread of current in the retina to just the neighboring electrodes, whereas a distant return electrode would allow a wider current spread. In theory, amongst these options, the hexapolar configuration has the greatest potential to limit the spread of activation across the two-dimensional retinal surface as it places a ring of “guard” electrodes around a central stimulating electrode. Consequently, it has received the greatest attention, and most studies have shown some benefit in using hexapolar over monopolar configurations in limiting the spread of neural activation. For example, patch recordings in *ex vivo* retina, Habib et al. (2013) showed that a hexapolar configuration limited the spread of retinal activation more than a monopolar configuration, with a pronounced increase in stimulation threshold outside the hex-guard that was not observed at equivalent distances in the monopolar configuration. Similarly, Spencer et al. (2016), found that a hexapolar sub-retinal configuration limited the spread of visual cortical activation for near threshold stimulation, when compared to monopolar stimulation. However, Cicione et al. (2012) found no significant difference in the spread of cortical activation between these two configurations, at least for stimulation levels approaching saturation. The difference between the studies of Spencer et al. and Cicione et al. may lie in the different stimulation level used to assess spread. Finally, concurrent stimulation with two adjacent hexapolar electrode configurations reduces or even eliminates crosstalk between them, but interference occurs when one or more of the two electrode configurations is monopolar. This has been demonstrated at the level of the electrical potential in saline *in vitro* (Dommel et al., 2009) as well as *in vivo* (Matteucci et al., 2016).

A potential limitation of the hexapolar configuration is that RGCs underlying the ring of sink electrodes may also be stimulated due to the relatively large currents entering those electrodes. Further, the sink electrodes may have very different impedances so that some electrodes will sink a larger fraction of the current than others when they are connected to a common ground. This will distort the area of activation toward electrodes sinking the largest fraction of current. To contend with these difficulties a focused multipolar approach (Figure 8E) has been proposed (Spencer et al., 2016). It overcomes the first limitation by using electrodes across the whole array to distribute the return current from a central stimulating electrode to optimally focus electrical potential. To overcome the second limitation relating to electrode impedance, it uses the implant to directly measure electrode impedances and correct for any distortion of the electrical potential they would cause. In practice this correction requires significant departures from a hexapolar configuration. Spencer et al. found that the area of visual cortex activated by focused multipolar and hexapolar sub-retinal stimulation was significantly reduced compared to monopolar sub-retinal stimulation, albeit at the cost of approximately 50% higher thresholds. However, no significant differences in activated areas were found between the focused multipolar and hexapolar configurations.

The hexapolar and multipolar approaches described above use combinations of electrodes as current sources and sinks to steer or focus current. An alternative approach, proposed by Spencer et al. (2019), is to shape retinal activity directly, rather than through current, by utilizing a model that predicts the pattern of retinal activity resulting from multielectrode stimulation, estimated from recordings made with the implant. The proposed stimulation strategy effectively inverts the model to find the pattern of electrical stimulation on the electrode array that optimally matches a target pattern of retinal activity. The strategy is most effective if all the electrodes on the array are used simultaneously to shape retinal activity, although in principle any number and configuration of electrodes can be optimized using the approach. An additional novel aspect of the strategy is that it shapes activity globally: the target pattern of retinal activity could cover any part of the retina spanned by the implant, and not just an isolated phosphene as considered in current focusing or steering strategies. Thus, it could represent the activity evoked in the retina by an entire image during sighted vision. When a focal phosphene is desired, the strategy can give similar solutions to the hexapolar and multipolar approaches (with the appropriate numbers and configurations of electrodes) provided the RGC response is not too heterogeneous across the array.

Accurate shaping of neural activity requires careful measurement of how multiple electrodes interact to produce a RGC response during simultaneous stimulation. *Ex vivo* retinal recordings to patterns of multielectrode stimulation have shown that for direct activation of RGCs, electrodes interact linearly during simultaneous stimulation in 90% of RGCs (Jepson et al., 2014a; Maturana et al., 2016). This conclusion is also supported by theoretical studies of multielectrode stimulation of biologically detailed models of RGCs based on morphological reconstruction with Hodgkin-Huxley type dynamics (Esler et al., 2018b). Following this, Maturana et al. (2016) showed that a model can accurately predict direct RGC responses to multielectrode stimulation if it is formulated in terms of an electrical receptive fields for each recorded RGC, which describes the contribution each electrode makes to stimulation of that cell in a linear weighted sum. The probability of the cell emitting a spike in response to multielectrode stimulation is a non-linear function of this weighted sum. For network mediated activation, a more complicated non-linear model is required (Maturana et al., 2018), although at the level of responses in visual cortex the simpler linear summation appears to suffice (Halupka et al., 2017a, b).

A key component of the strategy proposed by Spencer et al. (2019) is that it incorporates methods to determine the limitations on spatial resolution imposed by noise in the measurement of the RGC electrical receptive fields. Without noise, the strategy can in principle achieve a spatial resolution limited only by the spacing between electrodes. However, in practice, noise affects the higher spatial frequencies of the electrical receptive fields disproportionately, so that if the algorithm tries to use these spatial frequencies to optimize stimulation, gross departures from the target will result. The strategy can use the recordings from RGCs in response to multielectrode stimulation to identify the spatial frequencies at which noise exceeds the signal and use this to robustly optimize the spatial resolution of the implant.

## Future Directions

A significant amount of knowledge has been gained about the electrical stimulation of retinal prostheses using explanted retinas from animals. However, there is generally a lack of translation of the stimulation strategies developed *ex vivo* to clinical practice and it remains unclear whether they can improve the performance of retinal prostheses in patients. Some of the stimulation strategies in this review were developed using array configurations unavailable in clinic. Furthermore, most of the current research is conducted using healthy animals with normal vision. Several experimental results indicate that the retinal degeneration could introduce abnormal behavior in retinal neurons and their responses to electrical stimulation. Therefore, future research should focus more on the impact of degeneration.

In addition to searching for the optimal stimulation parameters for the spatiotemporal responses of populations of retinal neurons, it is now clear that retinal prostheses capable of simultaneous recording and stimulation will have the potential to significantly improve their performance via closed-loop feedback. The existing retinal prostheses available in the clinic can only stimulate. With no option of recording the neural activities from the retina, these devices can only rely on the feedback from patients to optimize their performance, which is very time consuming. The description from patients may be opaque, confusing, hard to quantify and vary according to their experiences and preferences. Also, regular device calibration will be necessary due to the changes in the electrode properties and retinal condition following implantation over time. An automatic adjustment using closed-loop feedback from the device can address the issue with much higher efficiency.

However, there are several challenges for the implementation of closed-loop retinal prostheses. First, current clinically available devices use electrodes with very large sizes, which are not suitable for high quality single-unit neural spike recording. To record from single neurons, electrodes around 10 μm will be necessary,

but such small electrodes create difficulties for neural stimulation, as described previously. It may be possible to combine several electrodes to provide sufficient stimulation capacity. Second, high quality neural spike recording will require a close contact between the electrodes and the target neurons, as the electrical potentials drop as a function of the square of the distances. Suprachoroidal and sub-retinal devices are both far away from the RGCs, while placement of epi-retinal devices close to the retinal surface has been a surgical challenge. Flexible electrode arrays are expected to be in better contact with the retinal surface than the rigid arrays, and might be a promising solution for neural recording. The third issue relates to data transmission and power. Single-unit recording normally requires signal sampling at a frequency of several tens of kilohertz. The amount of data that needs to be transmitted for external data analysis will be difficult considering the bandwidth for current technologies and will also consume a large amount of power. One strategy to reduce the data transmission is to incorporate the function of data processing into the implanted devices. However, such data processing will consume power and may generate a lot of heat that could be dangerous. One potential solution to solve all three problems is to replace the high frequency single unit recording with low frequency potential (LFP) recording, which records the collective activity of neural populations rather than the action potentials of each neuron. However, there has been very little work reported on LFP recordings in retina. It remains unclear if LFP recording can be used to study the responses of RGCs to electrical stimulation and how to use LFPs to inform the stimulation strategy.

## Conclusion

In the last few years, there has been a significant growth in research on the topic of electrical stimulation of retinal neurons, from both the basic understanding of the stimulation mechanisms to the development of novel stimulation strategies for better retinal prostheses performance. The research performed using explanted retinas from animals has provided insights on refining device efficiency by improving the spatial and temporal resolution possible from electrical stimulation, and has suggested potential approaches for selectively activating retinal neurons responsible for different visual processing. The next generation of retinal prostheses will benefit from the incorporation of neural recording, which is expected to further improve the overall performance based on closed-loop feedback.

## Author Contributions

All authors contributed to the writing of the manuscript.

## Conflict of Interest

DG was a shareholder and executive officer of Carbon Cybernetics Pty Ltd., a company developing diamond and carbon-based medical device components. The remaining authors declare that the research was conducted in the absence of any commercial or financial relationships that could be construed as a potential conflict of interest.
